# Safety and Efficacy of Camrelizumab in Combination With Nab-Paclitaxel Plus S-1 for the Treatment of Gastric Cancer With Serosal Invasion

**DOI:** 10.3389/fimmu.2021.783243

**Published:** 2022-01-18

**Authors:** Ju-Li Lin, Jian-Xian Lin, Jun Peng Lin, Chao-Hui Zheng, Ping Li, Jian-Wei Xie, Jia-bin Wang, Jun Lu, Qi-Yue Chen, Chang-Ming Huang

**Affiliations:** ^1^ Department of Gastric Surgery, Fujian Medical University Union Hospital, Fuzhou, China; ^2^ Department of General Surgery, Fujian Medical University Union Hospital, Fuzhou, China; ^3^ Key Laboratory of Ministry of Education of Gastrointestinal Cancer, Fujian Medical University, Fuzhou, China

**Keywords:** gastric cancer, camrelizumab (SHR-1210), neoadjuvant chemotherapy, tumor regression rate, pCR

## Abstract

**Objective:**

To investigate the safety and efficacy of camrelizumab in combination with nab-paclitaxel plus S-1 for the treatment of gastric cancer with serosal invasion.

**Method:**

Two hundred patients with gastric cancer with serosal invasion who received neoadjuvant therapy from January 2012 to December 2020 were retrospectively analyzed. According to the different neoadjuvant therapy regimens, the patients were divided into the following three groups: the SOX group (S-1 + oxaliplatin) (72 patients), SAP group (S-1 + nab-paclitaxel) (95 patients) and C-SAP group (camrelizumab + S-1 + nab-paclitaxel) (33 patients).

**Result:**

The pathological response (TRG 1a/1b) in the C-SAP group (39.4%) was not significantly different from that in the SAP group (26.3%) and was significantly higher than that in the SOX group (18.1%). The rate of ypT0 in the C-SAP group (24.2%) was higher than that in the SAP group (6.3%) and the SOX group (5.6%). The rate of ypN0 in the C-SAP group (66.7%) was also higher than that in the SAP group (38.9%) and the SOX group (36.1%). The rate of pCR in the C-SAP group (21.2%) was higher than that in the SAP group (5.3%) and the SOX group (2.8%). The use of an anti-PD-1 monoclonal antibody was an independent protective factor for TRG grade (1a/1b). The use of camrelizumab did not increase postoperative complications or the adverse effects of neoadjuvant therapy.

**Conclusion:**

Camrelizumab combined with nab-paclitaxel plus S-1 could significantly improve the rate of tumor regression grade (TRG 1a/1b) and the rate of pCR in gastric cancer with serosal invasion.

## Introduction

Gastric cancer is the fifth most common malignant tumor worldwide and the third leading cause of cancer-related death (1, 2). Surgical resection remains the only radical treatment available for patients with nonmetastatic gastric cancer. Because the recurrence rate remains high, multidisciplinary therapy, including neoadjuvant chemotherapy, has gradually become important for the treatment of advanced gastric cancer. In Europe and the Americas, docetaxel, oxaliplatin, fluorouracil, and leucovorin (the FLOT regimen) have become the standard neoadjuvant chemotherapy for advanced gastric cancer (CT2/N+M0) (3, 4). Compared with epirubicin, cisplatin, and fluorouracil or capecitabine (ECF/ECX regimen), the FLOT regimen has shown superiority in terms of pathological responses and overall survival outcomes. In China, the results of the RESOLVE trial (5) showed that the SOX regimen increased the overall survival rate of advanced gastric cancer (cT4aN+M0/cT4bN×M0) patients and the 3-year disease-free survival rate.

The KEYNOTE-059 (6) and ATTRACTION-2 (7) trials confirmed that PD-1 monoclonal antibody treatment provides significant survival benefit and good safety for advanced, recurrent or metastatic gastric/GEJ adenocarcinoma. Currently, the benefit of immunotherapy combined with neoadjuvant chemotherapy for locally advanced gastric cancer remains unclear. The safety and efficacy of immunotherapy in combination with neoadjuvant chemotherapy have not been reported in gastric cancer with serosal invasion. Therefore, the objective of this study was to investigate the safety and efficacy of camrelizumab in combination with nab-paclitaxel plus S-1 for the treatment of gastric cancer with serosal invasion.

## Methods

### Patient Selection

This study retrospectively analyzed the clinicopathological data of 200 patients who received SOX, nab-paclitaxel + S-1 or camrelizumab + nab-paclitaxel + S-1 neoadjuvant therapy and radical gastrectomy at the Fujian Union Hospital from January 2012 to December 2020. The inclusion criteria were as follows: gastric adenocarcinoma confirmed by gastroscopy and pathology before surgery; clinical stage: cT4, lymph node N1 to N3, nondistant metastasis (M0); ECOG score 0-2; and blood index, liver and kidney function, and cardiopulmonary function indicating that patients could tolerate chemotherapy or surgery. The exclusion criteria were as follows: distant metastasis or highly suspected metastasis; incomplete pathological diagnosis; gastric stump cancer; gastric cancer; emergency surgery; and combination with other malignant tumors.

### Neoadjuvant Therapy

We divided the patients into three groups according to the different neoadjuvant drug treatments: the SOX group (oxaliplatin + S-1), SAP group (nab-paclitaxel + S-1), and C-SAP group (camrelizumab + nab-paclitaxel + S-1). The specific scheme was as follows.

The cycle of SOX chemotherapy consisted of the following:

Day 1: Intravenous oxaliplatin 130 mg/m^2^

Days 1–14: S-1 at 120 mg/day for surface area ≥ 1.5 m², 100 mg/day for surface area between 1.25 and 1.5 m², and 80 mg/day for surface area < 1.25 m² were administered 2 times daily.

The next chemotherapy was repeated on the 22nd day.

The cycle of nab-paclitaxel + S-1 chemotherapy consisted of the following.

Day 1: Intravenous nab-paclitaxel 260 mg/m² over 30 min. Dose reductions (220 mg/m², 180 mg/m², or 150 mg/m²) were permitted in patients with severe hematological or nonhematological toxicity.

Days 1–14: S-1 at 120 mg/day for surface area ≥ 1.5 m², 100 mg/day for surface area between 1.25 and 1.5 m², and 80 mg/day for surface area < 1.25 m² were administered 2 times daily.

The next chemotherapy was repeated on the 22nd day.

The cycle of camrelizumab + nab-paclitaxel+S-1 chemotherapy consisted of the following.

Day 1: Intravenous camrelizumab 200 mg

Day 1: Intravenous nab-paclitaxel 260 mg/m² over 30 min. Dose reductions (220 mg/m², 180 mg/m², or 150 mg/m²) were permitted in patients with severe hematological or nonhematological toxicity.

Days 1–14: S-1 at 120 mg/day for surface area ≥ 1.5 m², 100 mg/day for surface area between 1.25 and 1.5 m², and 80 mg/day for surface area < 1.25 m² were administered 2 times daily.

The next chemotherapy was repeated on the 22nd day.

### Surgery

Patients underwent surgical resection between 2 and 4 weeks after the completion of neoadjuvant chemotherapy. Exploratory laparoscopy was routinely performed to exclude peritoneal or distant metastases. The scope of lymph node dissection was updated according to Japanese gastric cancer treatment guidelines (4th English edition) (8). TNM staging was performed according to the 8th edition of the AJCC/TNM staging system for gastric cancer (9).

### Study Endpoints

The primary endpoint of this study was the rate of tumor regression grade (TRG 1a/1b). The secondary end points included pCR, TNM stage, total number of lymph nodes, positive lymph nodes, complete (R0) resection rate, surgical complications, and neoadjuvant treatment-related adverse effects.

### Pathological Response

Tumor regression grade (TRG) was determined according to the Becker criteria (10, 11) and included “Grade 1a” (complete tumor regression, i.e., 0% residual tumor per tumor bed), “Grade 1b” (subtotal tumor regression, i.e., <10% residual tumor per tumor bed) “Grade 2” (partial tumor regression, i.e., 10–50% residual tumor per tumor bed), and “Grade 3” (minimal or no tumor regression, i.e., > 50% residual tumor per tumor bed).

Pathologic complete response (pCR) was defined as no invasive disease within submitted and evaluated gross lesions and histologically negative nodes based on central review.

### Tumor Staging

Radiologists followed the guidelines of the Response Evaluation Criteria in Solid Tumors (RECIST version 1.1) for the determination of radiological response to neoadjuvant chemotherapy (12). Two specialized radiologists independently evaluated the response rate, and the final result was determined after reviewing both sets of results.

### Neoadjuvant Therapy Cycles

CT evaluation was performed after 2 and 4 cycles of neoadjuvant therapy. Some patients could not tolerate the side effects of neoadjuvant therapy, so the neoadjuvant therapy cycle was less than 4 cycles. Some patients completed 4 cycles of neoadjuvant therapy. Because R0 resection could not be performed after CT evaluation, more cycles were added before surgery.

### Postoperative Complications

Postoperative complications were defined as events occurring within 30 days after the procedure, the severity of which was assessed by the Clavien-Dindo classification system (13, 14).

### Evaluation of Adverse Effects

Adverse effects were recorded according to the National Cancer Institute Common Terminology Criteria for Adverse Events (CTCAE 4.0). Drug dose or timing was adjusted for patients with grade three or worse adverse effects.

### Ethics

The study was approved by the ethics committee of Fujian Union Hospital. All of the patients signed informed consent documents.

### Statistical Methods

All of the data were analyzed by SPSS software (SPSS, Chicago, IL, USA), version 22.0. The chi-square test or Fisher’s exact test was used for comparisons of categorical variables. The independent sample t test or the Mann-Whitney U test was used for comparisons of continuous variables. Univariate logistic regression analysis was used to analyze the clinicopathological data of TRG (1a/1b). P < 0.05 was considered statistically significant.

## Results

### Clinical Characteristics

A total of 200 patients were included in this study, including the SOX group (72 patients), SAP group (95 patients) and C-SAP group (33 patients) (**Supplemental Figure 1**). There were no significant differences in age, sex, ECOG score, Baumann classification or tumor location among the three groups (all P > 0.05). The number of patients with tumor size > 5 cm was greater in the C-SAP group (51.5%) and the SAP group (48.4%) than in the SOX group (36.1%) (all P < 0.05). The proportion of poorly differentiated/undifferentiated tumors in the C-SAP group (69.7%) and the SAP group (63.2%) was higher than that in the SOX group (all P < 0.05). The proportion of patients with ≥ 4 cycles of preoperative neoadjuvant treatment in the SAP group (81.1%) was higher than that in the C-SAP group (66.7%) and the SOX group (36.1%) (all P < 0.05) (**Table 1**).

**Table 1:** Demographic data before surgery.

Baseline variable	C-SAP group (n = 33)	SAP group (n = 95)	P* value	SOX group (n = 72)	*P* ^#^ value	*P* ^&^ value
Gender			0.486		0.908	0.448
Male	26 (78.8)	69 (72.6)		56 (77.8)		
Female	7 (21.2)	26 (27.4)		16 (22.2)		
Age			0.988		0.962	0.969
< 60	13 (39.4)	31 (32.6)		27 (37.5)		
>= 60	20 (60.6)	64 (67.4)		45 (62.5)		
median	61.9 + 10.6	61.9 +12.3		62 + 10.8		
ECOG			0.530		0.379	0.724
0	29 (87.9)	87 (91.6)		67 (93.1)		
1	4 (12.1)	8 (8.4)		5 (6.9)		
Tumor size						
<= 5 cm	16 (48.5)	49 (51.6)	0.550	46 (63.9)	0.01	0.03
> 5 cm	17 (51.5)	46 (48.4)		26 (36.1)		
median (cm)	5.5	5.1		4.8		
Baumann type			0.786		0.214	0.354
2-3	26 (78.8)	73 (76.8)		59 (81.9)		
4	7 (21.2)	22 (23.2)		13 (18.1)		
Neoadjuvant cycle			0.010		0.001	0.001
<= 3	11 (33.3)	18 (18.9)		46 (63.9)		
>= 4	22 (66.7)	77 (81.1)		26 (36.1)		
Tumor location			0.530		0.379	0.724
Upper	18 (54.5)	46 (48.4)		37 (51.4)		
Middle	10 (30.3)	22 (23.2)		19 (26.4)		
Lower	5 (15.2)	27 (28.4)		16 (22.2)		
Differentiation			0.235		0.001	0.001
Well and middle	10 (30.3)	35 (36.8)		37 (51.4)		
Poor and underdifferentiated	23 (69.7)	60 (63.2)		35 (48.6)		

P^*^: C-SAP vs. SAP P^#^: C-SAP vs. SOX P^&^: SAP vs. SOX.

### Pathological Response

There was no significant difference in the rate of TRG grade (1a + 1b) between the C-SAP group (39.4%) and the SAP group (26.3%) (P > 0.05), but the rate in these two groups was higher than that in the SOX group (18.1%) (P < 0.05). The proportion of ypT0 in the C-SAP group (24.2%) was higher than that in the SAP group (6.3%) and the SOX group (5.6%). The proportion of ypN0 in the C-SAP group (66.7%) was higher than that in the SAP group (38.9%) and the SOX group (36.1%). The proportion of pCR in the C-SAP group (21.2%) was higher than that in the SAP group (5.3%) and the SOX group (2.8%) (both P < 0.05). There was no significant difference in the proportion of TRG grade (1a + 1b), the proportion of ypT0, the proportion of ypN0 or the proportion of pCR between the SAP group and the SOX group (P> 0.05) (**Table 2**). **Supplemental Figure 3** shows the effects of different neoadjuvant chemotherapy regimens and cycles on TRG in detail. The rate of TRG (1a + 1b) of patients receiving ≥ 4 cycles of neoadjuvant therapy group was higher than that of patients receiving ≤ 3 cycles of neoadjuvant therapy in C-SAP; The rate of TRG (1a + 1b) of patients receiving ≥ 4 cycles of neoadjuvant therapy was lower than that of patients receiving ≤ 3 cycles of neoadjuvant therapy in the SAP and SOX groups.

**Table 2:** Differences in response among the three groups.

Baseline variable	C-SAP group (n = 33)	SAP group (n = 95)	P* value	SOX group (n = 72)	*P* ^#^ value	*P* ^&^ value
TRG			0.034		0.029	0.543
TRG1a	8 (24.2)	6 (6.3)		4 (5.6)		
TRG1b	5 (15.2)	19 (20.0)		9 (12.5)		
TRG2	6 (18.2)	32 (33.7)		24 (33.3)		
TRG3	14 (42.4)	38 (40.0)		35 (48.6)		
subgroup analysis			0.157		0.019	0.207
TRG1a-1b	13 (39.4)	25 (26.3)		13 (18.1)		
TRG2-3	20 (60.6)	70 (73.7)		59 (81.9)		
ypTstage			0.027		0.078	0.039
T0	8 (24.2)	6 (6.3)		4 (5.6)		
T1	2 (6.1)	11 (11.6)		4 (5.6)		
T2	4 (12.1)	12 (12.6)		5 (6.9)		
T3	13 (39.4)	55 (57.9)		37 (51.4)		
T4a	5 (15.2)	11 (11.6)		21 (29.2)		
T4b	1 (3.0)	0		1 (1.4)		
ypNstage			0.055		0.056	0.563
N0	22 (66.7)	37 (38.9)		26 (36.1)		
N1	5 (15.2)	21 (22.1)		14 (19.4)		
N2	2 (6.1)	15 (15.8)		12 (16.7)		
N3a	2 (6.1)	17 (17.9)		11 (15.3)		
N3b	2 (6.1)	5 (5.3)		9 (12.5)		
ypTNMstage			0.015		0.003	0.504
pCR	7 (21.2)	5 (5.3)		2 (2.8)		
I	6 (18.2)	17 (17.9)		9 (12.5)		
II	12 (36.4)	35 (36.8)		25 (34.7)		
III	8 (24.2)	38 (40.0)		36 (50.0)		
ypTstage			0.004		0.014	1
T0	8 (24.22)	6 (6.3)		4 (5.6)		
T1-T4b	25 (75.8)	89 (93.7)		68 (94.4)		
ypNstage			0.006		0.004	0.708
N0	22 (66.7)	37 (38.9)		26 (36.1)		
N1-N3b	11 (33.3)	58 (61.1)		46 (63.9)		
ypTstage			0.006		0.002	0.686
pCR	7 (21.2)	5 (5.3)		2 (2.8)		
I-III	26 (78.8)	90 (94.7)		70 (97.2)		
Radiological response			0.754		0.918	0.587
PR	30 (90.9)	88 (92.6)		65 (90.3)		
SD	3 (9.1)	7 (7.4)		7 (9.7)		

P^*^: C-SAP vs. SAP P^#^: C-SAP vs. SOX P^&^: SAP vs. SOX.

### Risk Factors for Pathological Response

By univariate analysis, we found that the influencing factors of TRG (1a + 1b) included tumor size, Baumann classification, the use of PD-1, tumor location and pathological differentiation type. The multivariate analysis showed that tumor size > 5 cm was an independent risk factor (OR = 3.791, 95% CI = 1.513-9.501, P = 0.004), while the use of PD-1 was an independent protective factor (OR = 0.36, 95% CI = 0.152-0.852, P = 0.02) (**Supplemental Table 1**). By univariate analysis, we found that the factors influencing lymph node staging (ypN0) included tumor size, Baumann classification, tumor location and the use of PD-1. The multivariate analysis showed that middle gastric cancer was an independent risk factor (OR = 3.653, 95% CI = 1.163-8.275, P = 0.002), while the use of PD-1 was an independent protective factor (OR = 0.215, 95% CI = 0.88-0.525, P = 0.001) (**Supplemental Table 2**).

### Comparison of Postoperative Conditions

There were no significant differences in the type of gastrectomy, surgical approach, R0 resection, nerve invasion or vascular invasion among the three groups (all P > 0.05). There were no significant differences in the number of harvested lymph nodes and positive lymph nodes between the C-SAP and SAP groups (all P > 0.05). The number of harvested lymph nodes in the C-SAP group was greater than that in the SOX group, and the number of positive lymph nodes in the C-SAP group was lower than that in the SOX group (all P < 0.05) (**Supplemental Figure 2**). In the SAP group, the treatment of 1 patient (1.1%) was combined with partial hepatectomy. In the SOX group, the treatment of 1 patient (1.4%) was combined with transverse colectomy, and 1 patient (1.4%) received body and tail pancreatectomy (**Table 3**).

**Table 3:** Clinicopathological results after surgery.

Baseline variable	C-SAP group (n = 33)	SAP group (n = 95)	P* value	SOX group (n = 72)	P^#^ value	P^&^ value
Type of gastrectomy			0.44		0.503	0.493
Partial	4 (12.4)	17 (17.9)		14 (19.4)		
Total	29 (87.9)	78 (82.1)		58 (80.6)		
Surgical approach			0.109		1.000	0.156
Laparoscopy	31 (93.9)	95 (100)		69 (95.8)		
Open	2 (6.1)	0		3 (4.2)		
Combination organ dissection						
Transverse colon				1 (1.4)		
Body and tail of pancreas				1 (1.4)		
Partial left liver		1 (1.1)				
Extent of resection			0.578		0.568	0.889
R0	32 (97.0)	94 (98.9)		68 (94.4)		
R1	1 (3.0)	1 (1.1)		4 (5.6)		
Nerve invasion			0.144		0.924	0.048
No	25 (75.8)	58 (61.7)		51 (71.8)		
Yes	8 (24.2)	36 (38.3)		20 (28.2)		
Vessel invasion			0.107		0.506	0.3
No	24 (72.7)	54 (56.8)		50 (69.4)		
Yes	9 (27.3)	41 (432.2)		22 (30.6)		
Harvested lymph nodes			0.569		0.039	< 0.001
Median	41.3 ± 19.3	43.1 ± 14.0		34.8 ± 10.5		
Positive lymph nodes			0.124		0.033	0.517
Median	2.2 ± 5.2	4.3 ± 6.9		4.9 ± 6.6		

P*, C-SAP vs. SAP; P^#^, C-SAP vs. SOX; P^&^, SAP vs. SOX.

### Postoperative Complications

The overall complication rate was 25.5%, the Grade II complication rate was 22.5%, the Grade III complication rate was 3.5%, and there were no Grade IV or V complications.

There were no significant differences in the proportions of postoperative complications among the three groups (C-SAP (24.2%), SAP (22.1%) and SOX (31.9%)) (P > 0.05). The proportion of Grade II complications was 18.2% in the C-SAP group, 18.9% in the SAP group and 29.2% in the SOX group, with no significant differences (P > 0.05). The proportion of Grade III complications was 6.1% in the C-SAP group, 3.2% in the SAP group and 2.8% in the SOX group, with no significant differences (P > 0.05). There were no significant differences in the rates of pneumonia, abdominal infection, postoperative bleeding or anastomotic leakage among the three groups (P > 0.05) (**Table 4**).

**Table 4:** Postoperative complications.

Baseline variable	C-SAP group (n = 33)	SAP group (n = 95)	P* value	SOX group (n = 72)	P^#^ value	P^&^ value
Postoperative complications (yes)	8 (24.2)	21 (22.1)	0.801	23 (31.9)	0.422	0.153
Clavien Dindo grading						
Grade I-II	6 (18.2)	18 (18.9)	0.923	21 (29.2)	0.232	0.122
Pulmonary infection	5 (15.2)	14 (14.7)	0.954	16 (21.3)	0.455	0.263
Abdominal infection	1 (3.0)	4 (4.2)	1.000	5 (11.3)	0.761	0.715
Grade III	2 (6.1)	3 (3.2)	0.826	2 (2.8)	0.790	1.000
Bleeding	1 (3.0)	1 (1.1)	1.000	1 (1.3)	1.000	1.000
Obstruction	0	0	NA	1 (1.3)	> 0.99	> 0.99
Anastomotic leakage	1 (3.0)	2 (2.1)	1	0	> 0.99	> 0.99
Grade IV	0	0	NA	0	NA	NA
Grade V	0	0	NA	0	NA	NA

P*, C-SAP vs. SAP; P^#^, C-SAP vs. SOX; P^&^, SAP vs. SOX. NA, Not applicable.

### Adverse Effects Associated With Neoadjuvant Therapy

We analyzed the adverse effects associated with neoadjuvant therapy. The most common adverse effects (Grades 3 and 4) were decreased WBC count, decreased neutrophil count and increased serum AST/ALT ratio. Neutrophil count decreased (Grades 3, 4) in the C-SAP group (24.8%) and the SAP group (23.2%) and were significantly higher than in the SOX group (9.7%) (P < 0.05), but there was no significant difference between the C-SAP group and the SAP group (P>0.05). WBC decreased (Grade s3 and4) in the C-SAP group (15.2%) and the SAP group (21.1%) and was higher than in the SOX group (5.6%), but there was no significant difference between the C-SAP group and the SAP group (P > 0.05). Serum AST/ALT increased in the C-SAP group (24.2%), SAP group (12.6%) and SOX group (20.8%), and there were no significant differences among the three groups (P > 0.05). The levels of anemia (Grades 3 and 4) in the C-SAP group (3.0%), SAP group (2.1%) and SOX group (6.9%) (P>0.05) were not significantly different. The platelet count was decreased (Grades 3 and 4) in the C-SAP group (6.1%), SAP group (3.2%) and SOX group (5.6%), and there was no significant difference among the three groups (P > 0.05). There was no significant difference in the rate of febrile neutropenia among the C-SAP (3.0%), SAP (5.3%) and SOX (2.8%) groups (P > 0.05) (**Table 5**).

**Table 5:** Neoadjuvant treatment adverse effects.

Baseline Variable	C-SAP group (n=33)	SAP group (n=95)	P* value	SOX group (n=72)	P^#^ value	P^&^ value
WBC decreased			0.461		0.103	0.005
Grade 0, 1, 2	28 (84.8)	75 (78.9)		68 (94.4)		
Grade 3, 4	5 (15.2)	20 (21.1)		4 (5.6)		
Neutrophil count decreased			0.899		0.048	0.023
Grade 0, 1, 2	25 (75.8)	73 (76.8)		65 (90.3)		
Grade 3, 4	8 (24.8)	22 (23.2)		7 (9.7)		
Anemia			1.000		0.422	0.122
Grade 0, 1, 2	32 (97.0)	93 (97.9)		67 (93.1)		
Grade 3, 4	1 (3.0)	2 (2.1)		5 (6.9)		
Platelet count decreased			0.458		0.918	0.444
Grade 0, 1, 2	31 (93.9)	92 (96.8)		68 (94.4)		
Grade 3, 4	2 (6.1)	3 (3.2)		4 (5.6)		
Serum AST/ALT increase			0.114		0.695	0.154
Normal	25 (75.8)	83 (87.4)		57 (79.2)		
Increase	8 (24.2)	12 (12.6)		15 (20.8)		
Febrile neutropenia			0.601		1.000	0.427
No	32 (97.0)	90 (94.7)		70 (97.2)		
Yes	1 (3.0)	5 (5.3)		2 (2.8)		

P*, C-SAP vs SAP; P^#^, C-SAP vs SOX; P^&^, SAP vs SOX.

## Discussion

The safety and efficacy of immunotherapy in combination with neoadjuvant chemotherapy have not been reported in gastric cancer with serosal invasion. This study is the first to investigate the safety and efficacy of camrelizumab in combination with nab-paclitaxel plus S-1 for the treatment of gastric cancer with serosal invasion. The results showed that camrelizumab in combination with neoadjuvant chemotherapy significantly increased the rate of tumor regression grade (TRG grade 1a/1b) and the rate of pCR in gastric cancer with serosal invasion and did not increase postoperative complications or neoadjuvant treatment-related adverse effects.

For patients receiving neoadjuvant chemotherapy, tumor regression grade is an important factor affecting the overall survival rate (11). The SOX regimen is a commonly used neoadjuvant chemotherapy regimen in the Asian population. A large-scale, randomized, controlled trial (RESOLVE) (5) from China showed that the SOX regimen has good application prospects as a neoadjuvant chemotherapy for advanced gastric cancer. The results of a phase II clinical trial (Dragon III) (15) revealed that the rate of tumor regression grade (TRG grade 1a/1b) in locally advanced gastric cancer (cT4/NxM0) treated with the SOX regimen was 32.4%. A nab-paclitaxel regimen and camrelizumab combined with a nab-paclitaxel regimen in the treatment of locally advanced gastric cancer have not been reported. In this study, the rate of tumor regression grade (TRG grade 1a/1b) in the C-SAP group (39.4%) was not significantly different from that in the SAP group (26.3%) but was significantly higher than that in the SOX group (18.1%). Therefore, camrelizumab combined with neoadjuvant chemotherapy could increase the tumor regression grade (TRG grade 1a/1b).

Pathological complete response (pCR) has been shown to correlate with overall survival (OS) outcomes (16). More than ten studies focusing on immunotherapy combined with neoadjuvant chemotherapy for the treatment of gastric cancer have been conducted, most of which evaluated pCR as the primary endpoint. The rate of pCR was 22.2% (2/9) when sintilimab was combined with the FLOT regimen for the neoadjuvant treatment of gastric or gastroesophageal junction (GEJ) adenocarcinoma (17). The rate of pCR was 23.1 (6/26) when sintilimab plus oxaliplatin/capecitabine (CapeOx) was used as a neoadjuvant therapy for patients with locally advanced, resectable gastric (G)/esophagogastric junction (GEJ) adenocarcinoma (18). The rate of pCR was 8% (2/26) when camrelizumab was combined with FOLFOX as a neoadjuvant therapy for resectable locally advanced gastric and gastroesophageal junction adenocarcinoma (19). In this study, the rate of pCR in the C-SAP group was 21.2% (7/33), similar to that observed in previous studies, and it was significantly higher than that in the SAP group (5.3%, 5/95) and the SOX group (2.8%, 2/72). Therefore, the results of this study showed that camrelizumab combined with neoadjuvant chemotherapy could improve the rate of pCR.

In the Dragon III study (15), univariate analyses showed that TRG was correlated with sex, nerve invasion, vascular invasion and postoperative pathological stage. However, the general clinical data of the three groups of patients in this study were not balanced. The number of patients with tumor size > 5 cm in the C-SAP group (51.5%) and the SAP group (48.4%) was higher than that in the SOX group (36.1%). The number of patients with poorly differentiated/undifferentiated tumors in the C-SAP group (69.7%) and the SAP group (63.2%) was higher than that in the SOX group (all P < 0.05). However, the multivariate analysis showed that tumor size > 5 cm was an independent risk factor, while the use of camrelizumab was an independent protective factor.

The postoperative safety of patients after neoadjuvant therapy remains unclear. Li et al (20) reported that laparoscopic distal gastrectomy appeared to offer better postoperative safety than open distal gastrectomy for patients with locally advanced gastric cancer who received neoadjuvant chemotherapy. Li et al. reported that the LADG group was less likely to have Clavien-Dindo Grade II complications than the ODG group (6 [13%] vs. 20 [40%]; P = 0.004). Six patients (13%) in the LADG group and 2 patients (4%) in the ODG group had Grade III or higher complications (P = 0.25). Only 1 patient (2%) in the laparoscopic group had Grade IV complications, and no Grade V complications were reported. In our study, the overall complication rate of the whole group was 25.5%, the complication rate of Grade II events was 22.5%, and the complication rate of Grade III events was 3.5%. There were no Grade IV or V events reported, similar to the findings reported by Li et al. Additionally, the rate of Grade II complications was 18.2% in the C-SAP group, 18.9% in the SAP group and 29.2% in the SOX group. The rate of Grade III complications was 6.1% in the C-SAP group, 3.2% in the SAP group and 2.8% in the SOX group. Therefore, camrelizumab combined with neoadjuvant chemotherapy did not increase postoperative complications.

The WBC decreased (Grades 3, 4) by 41% in patients treated weekly with SAP in the ABSOLUTE trial (21). There was no significant difference in terms of WBC decreases (Grades 3 and 4) among the three groups in our study. There was no significant difference in terms of neutrophil count decreases (Grades 3 and 4) between the C-SAP and SAP groups. In this study, only one patient suffered febrile neutropenia (3%) in the C-SAP group, similar to that observed in the weekly SAP group (3%) in the ABSOLUTE trial, while there was no significant difference in terms of febrile neutropenia among the three groups. Therefore, camrelizumab combined with neoadjuvant chemotherapy is safe.

Although there was no significant difference in TRG between the C-SAP and SAP groups, we found that the rate of TRG (1a + 1b) in the C-SAP group was higher than that in the SAP group. Camrelizumab might be the reason for the difference between the two groups. Therefore, for gastric cancer patients with cT4a/T4b, the use of camrelizumab might predict a higher rate of TRG (1a + 1b). In addition, the rates of yPCR, yT0 and yN0 in the C-SAP group were significantly higher than those in the SAP group. The reason for the difference was the use of camrelizumab.

The optimal cycles of neoadjuvant therapy are controversial. A possible reason is that patients with obvious tumor regression receive 3-4 cycles of neoadjuvant therapy before surgery. Patients with no obvious tumor regression were asked to receive more cycles on the basis of 3-4 cycles. For patients in the SAP and SOX groups, the rate of TRG (1a + 1b) of patients receiving ≥ 4 cycles of neoadjuvant therapy was lower than that receiving ≤ 3 cycles of neoadjuvant therapy in the SAP and SOX groups. Therefore, the optimal neoadjuvant therapy cycle for these patients is 3 cycles. For patients in the C-SAP group, the rate of TRG (1a + 1b) of patients receiving ≥ 4 cycles of neoadjuvant therapy was higher than that of patients receiving ≤ 3 cycles of neoadjuvant therapy. Therefore, the optimal neoadjuvant therapy cycle for these patients is 4 cycles.

There are several limitations of this study. First, this study was a real-world, retrospective study with selection bias. Our center did not use the chemotherapy regimen of camrelizumab + SOX. Second, the number of patients in the C-SAP group was relatively small. PD-L1 expression was not measured, and differences in CPS could have affected the results of this study. Most of the patients in this study were followed up for less than 3 years.

In conclusion, camrelizumab in combination with nab-paclitaxel plus S-1 could significantly improve the rate of tumor regression grade (TRG 1a/1b) and the rate of pCR in gastric cancer with serosal invasion and did not increase postoperative complications or neoadjuvant treatment-related adverse effects. The results of this study must be further confirmed by prospective, randomized, controlled trials.

## Data Availability Statement

The raw data supporting the conclusions of this article will be made available by the authors, without undue reservation.

## Ethics Statement

The studies involving human participants were reviewed and approved by the ethics committee of Fujian Union Hospital. The patients/participants provided their written informed consent to participate in this study.

## Author Contributions

Study concepts, statistical analysis, data analysis, and interpretation, manuscript preparation, and manuscript editing: J-LL, J-XL, JPL, C-MH. Study design, data acquisition, quality control of data, and algorithms: J-LL, J-XL, JPL, C-HZ, PL, J-WX, J-BW, JL, Q-YC, C-MH. All authors contributed to the article and approved the submitted version.

## Funding

The second batch of special support funds for Fujian Province innovation and entrepreneurship talents (2016B013).

## Conflict of Interest

The authors declare that the research was conducted in the absence of any commercial or financial relationships that could be construed as a potential conflict of interest.

## Publisher’s Note

All claims expressed in this article are solely those of the authors and do not necessarily represent those of their affiliated organizations, or those of the publisher, the editors and the reviewers. Any product that may be evaluated in this article, or claim that may be made by its manufacturer, is not guaranteed or endorsed by the publisher.

## Supplementary Material

Supplementary Figure 1 Diagram.

Supplementary Figure 2 There was no significant difference in terms of the total number of lymph nodes and positive lymph nodes between the C-SAP and SAP groups (all P > 0.05). However, the total number of lymph nodes in the C-SAP group was greater than that in the SOX group, and the number of positive lymph nodes in the C-SAP group was less than that in the SOX group (all P < 0.05).

Supplementary Figure 3 Different neoadjuvant chemotherapy regimens and cycles on TRG.

## Abbreviations

S-1, tegafur gimeracil oteracil potassium capsule; TRG, tumor regression grade; pCR, pathological complete response; the FLOT regimen, docetaxel, oxaliplatin, fluorouracil, and leucovorin; ECF/ECX regimen, epirubicin, cisplatin, and fluorouracil or capecitabine.
