# Supplementary figures and images for: Safety and Efficacy of Camrelizumab in Combination With Nab-Paclitaxel Plus S-1 for the Treatment of Gastric Cancer With Serosal Invasion
Source: Front Immunol. 2022 Jan 18;12:783243. doi: 10.3389/fimmu.2021.783243 (PMC8805791; doi:10.3389/fimmu.2021.783243)

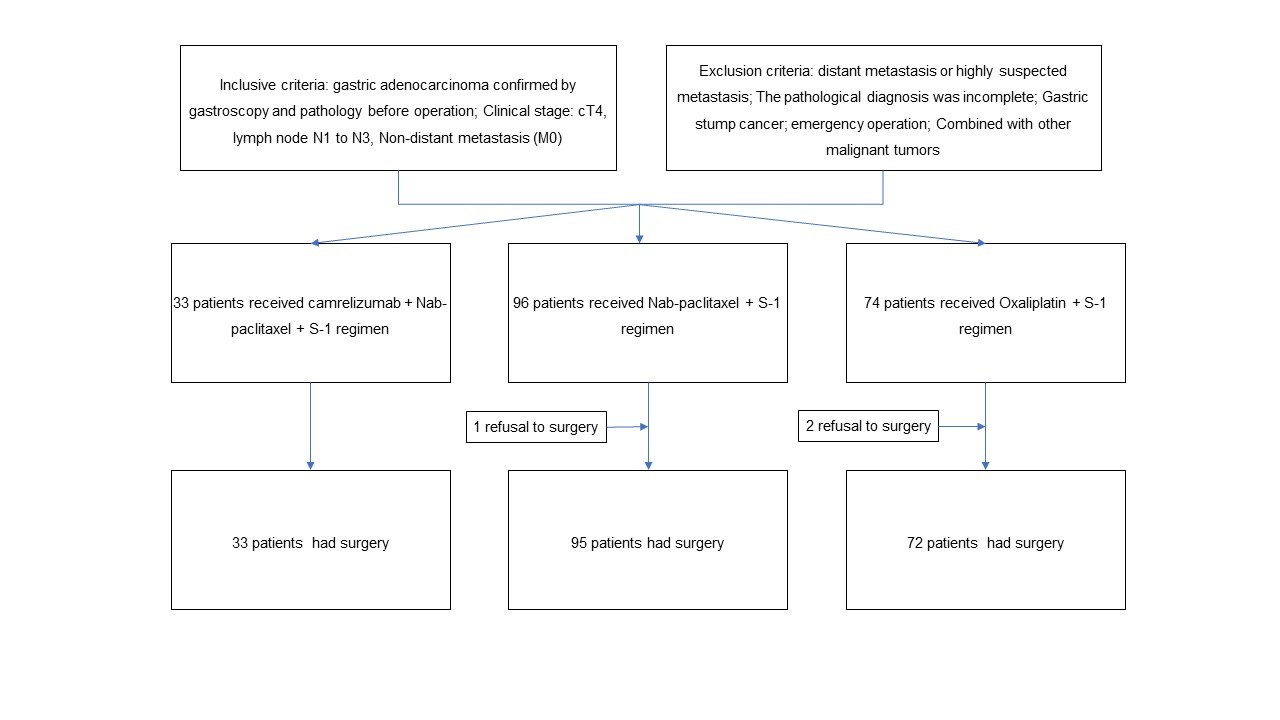

Supplement: Supplementary Figure 1 — Diagram. [file Image_1.jpeg]

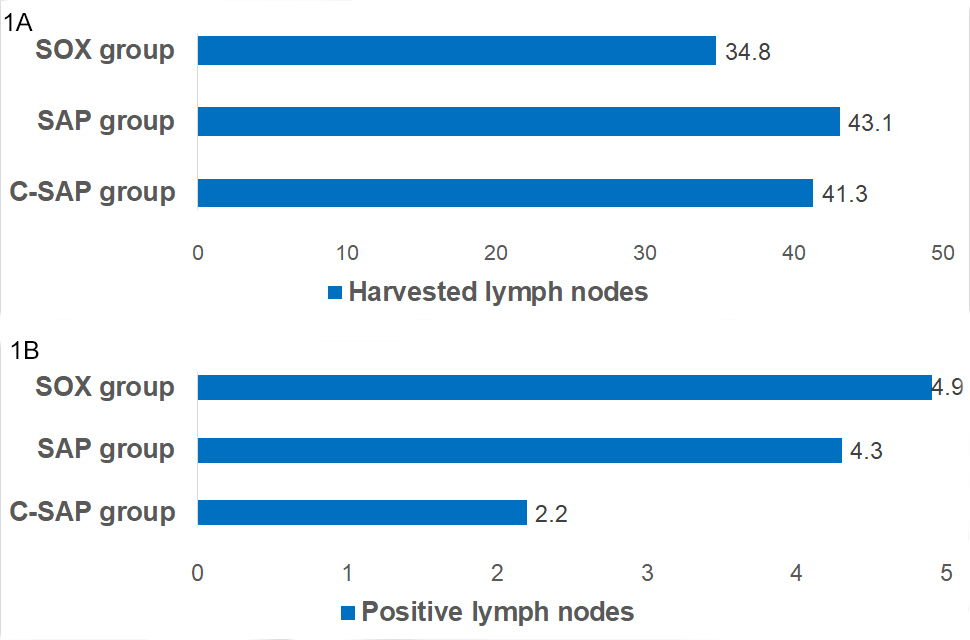

Supplement: Supplementary Figure 2 — There was no significant difference in terms of the total number of lymph nodes and positive lymph nodes between the C-SAP and SAP groups (all P > 0.05). However, the total number of lymph nodes in the C-SAP group was greater than that in the SOX group, and the number of positive lymph nodes in the C-SAP group was less than that in the SOX group (all P < 0.05). [file Image_2.jpeg]

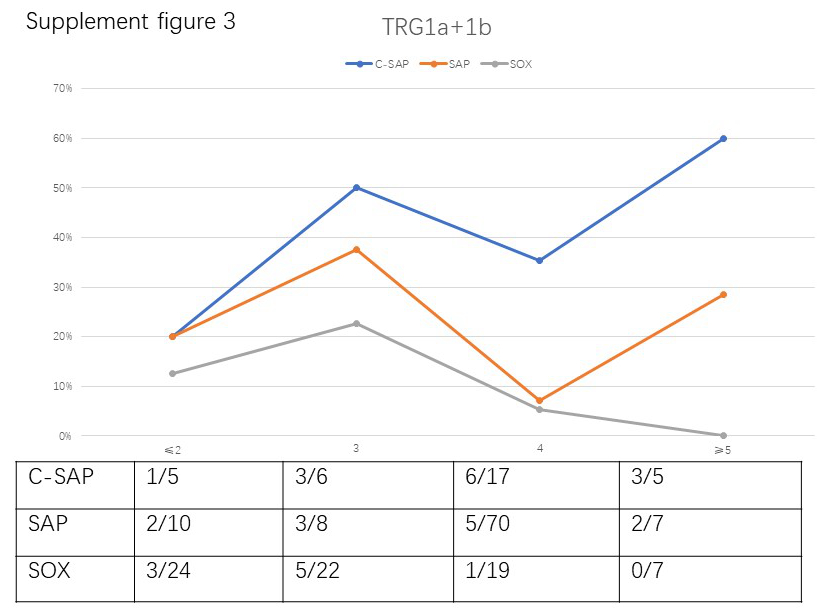

Supplement: Supplementary Figure 3 — Different neoadjuvant chemotherapy regimens and cycles on TRG. [file Image_3.jpeg]
