# Supplementary material for: Safety and Efficacy of Camrelizumab in Combination With Nab-Paclitaxel Plus S-1 for the Treatment of Gastric Cancer With Serosal Invasion
Source: Front Immunol. 2022 Jan 18;12:783243. doi: 10.3389/fimmu.2021.783243 (PMC8805791; doi:10.3389/fimmu.2021.783243)
Supplement: Supplementary file 4 [file Table_1.docx]

| Supplement Table 1 Univariable and multivariable analyses of clinicopathologic variables in relation to TRG | | | | | | |
| --- | --- | --- | --- | --- | --- | --- |
| Baseline Variable | TRG1a-1b (n=51) | TRG2-3 (n=149) | P value | RR | (95% CI) | P* value |
| Gender |  |  | 0.852 |  |  |  |
| male | 39(76.5) | 112(75.2) |  |  |  |  |
| female | 12(23.5) | 37(24.8) |  |  |  |  |
| Age |  |  | 0.708 |  |  |  |
| <60 | 17(33.3) | 54(36.2) |  |  |  |  |
| >=60 | 34(66.7) | 95(63.8) |  |  |  |  |
| Tumor size |  |  | <0.001 |  |  |  |
| <=5cm | 41(80.4) | 70(47.0) |  | Ref |  |  |
| >5cm | 10(19.6) | 79(53.0) |  | 3.791 | 1.513-9.501 | 0.004 |
| Baumann type |  |  | 0.004 |  |  |  |
| 2-3 | 48(94.1) | 110(73.8) |  | Ref |  |  |
| 4 | 3(5.9) | 39(26.2) |  | 2.186 | 0.519-9.203 | 0.286 |
| Neoadjuvant cycle |  |  | 0.476 |  |  |  |
| <=3 | 17(33.3) | 58(38.9) |  |  |  |  |
| >=4 | 34(66.7) | 91(61.1) |  |  |  |  |
| Camrelizumab |  |  | 0.045 |  |  |  |
| No | 38(74.5) | 129(86.6) |  | Ref |  |  |
| Yes | 13(25.5) | 20(13.4) |  | 0.36 | 0.152-0.852 | 0.02 |
| Neoadjuvant treatment |  |  | 0.07 |  |  |  |
| SAP | 38(74.5) | 90(60.4) |  |  |  |  |
| SOX | 13(25.5) | 59(39.6) |  |  |  |  |
| Tumor location |  |  | 0.196 |  |  |  |
| Upper | 31(60.8) | 70(47.0) |  |  |  |  |
| Middle | 9(17.6) | 42(28.2) |  |  |  |  |
| Lower | 11(21.6) | 37(24.8) |  |  |  |  |
| Differentiation |  |  | 0.308 |  |  |  |
| Well and middle | 24(47.1) | 58(38.9) |  |  |  |  |
| Poor and underdifferentiated | 27(52.9) | 91(61.1) |  |  |  |  |

| Supplement Table 2 Univariable and multivariable analyses of clinicopathologic variables correlation to ypN0 | | | | | | |
| --- | --- | --- | --- | --- | --- | --- |
| Baseline Variable | ypN0 (n=85) | ypN1-3b (n=115) | P value | RR | (95% CI) | P* value |
| Gender |  |  | 0.065 |  |  |  |
| male | 24(28.2) | 47(40.9) |  |  |  |  |
| female | 61(71.8) | 68(59.1) |  |  |  |  |
| Age |  |  | 0.708 |  |  |  |
| <60 | 59(69.4) | 92(80.0) |  |  |  |  |
| >=60 | 26(30.6) | 23(20.0) |  |  |  |  |
| Tumor size |  |  | 0.011 |  |  |  |
| <=5cm | 56(65.9) | 55(47.8) |  | Ref |  |  |
| >5cm | 29(34.1) | 60(52.2) |  | 1.669 | 0.783-3.557 | 0.185 |
| Baumann type |  |  | 0.002 |  |  |  |
| 2-3 | 76(89.4) | 82(71.3) |  | Ref |  |  |
| 4 | 9(10.6) | 33(28.7) |  | 2.074 | 0.758-5.674 | 0.155 |
| Neoadjuvant cycle |  |  | 0.083 |  |  |  |
| <=3 | 26(30.6) | 49(42.6) |  |  |  |  |
| >=4 | 59(69.4) | 66(57.4) |  |  |  |  |
| PD-1 |  |  | 0.002 |  |  |  |
| No | 63(74.1) | 104(90.4) |  | Ref |  |  |
| Yes | 22(25.9) | 11(9.6) |  | 0.215 | 0.88-0.525 | 0.001 |
| Neoadjuvan treatment | |  | 0.17 |  |  |  |
| Abraxane | 59(69.4) | 69(60.0) |  |  |  |  |
| SOX | 26(30.6) | 46(40.0) |  |  |  |  |
| Tumor location |  |  | 0.002 |  |  |  |
| Upper | 54(63.5) | 47(40.9) |  | Ref |  | 0.006 |
| Middle | 12(14.1) | 39(33.9) |  | 3.653 | 1.613-8.275 | 0.002 |
| Lower | 19(22.4) | 29(25.2) |  | 1.785 | 0.846-3.769 | 0.128 |
| Differentiation |  |  | 0.038 |  |  |  |
| well | 42(49.4) | 40(34.8) |  | Ref |  |  |
| poor | 43(50.6) | 75(65.2) |  | 1.841 | 0.977-3.469 | 0.059 |

Supplement table 3 The similarity and differences between this study and other studies

| Study | Dedign | Group | Regimen | Primary endpoint | Population | Sample size | Period | PD-1/PD-L1 |
| --- | --- | --- | --- | --- | --- | --- | --- | --- |
| Our study | Retrospective | Three group | Neo C-SAP/  Neo SAP/  Neo SOX | TRG(1a+1b) | Chinese  Advanced gastric cancer | 33 / 95 / 72 | 2012-2020 | Camrelizumab |
| RESOLVE trial  (NCT01534546) | Prospective | Three group | Peri SOX/  Adj SOX/  Adj CapeOX | 3 years DFS | Chinese  Advanced gastric cancer | 337 / 340 / 345 | 2012-2021 | NON |
| Dragon III trial  (NCT03636893) | Prospective | Two group | Neo FLOT/  Neo SOX | TRG(1a+1b) | Chinese  Advanced gastric cancer | 40 / 34 | 2018-2020 | NON |
| ABSOLUTE trial  (JapicCTI-132059) | Prospective | Three group | Nab-paclitaxel every 3 weeks/  nab-paclitaxel weekly/  solvent-based paclitaxel weekly | Overall survival | Japan  Advanced gastric cancer | 247 / 246 / 248 | 2013-2015 | NON |
| KEYNOTE-659  (NCT03382600) | Prospective | Two group | Neo SOX/  Neo SP | ORR | Japan  Advanced gastric cancer | 45 / 45 | 2018-2021 | Pembrolizumab |
| ATTRACTION-4  (NCT02746796) | Prospective | Three group | Nivolumab plus SOX /  Nivolumab plus CapeOX /  Placebo plus sox OR CapeOX | Progression-free survival | Japan  Advanced or Metastatic Gastric cancer | 680 | 2016-2022 | Nivolumab |
| CheckMate-649  (NCT02872116) | Prospective | Four group | Nivolumab plus XELOX/  Nivolumab plus CapeOX /  XELOX / CapeOX | Overall survival | Global Advanced or Metastatic Gastric cancer | / | 2016-2021 | Nivolumab |
